# Effect of sex and fatigue on single leg squat kinematics in healthy young adults

**DOI:** 10.1186/s12891-015-0739-3

**Published:** 2015-09-30

**Authors:** Benjamin K. Weeks, Christopher P. Carty, Sean A. Horan

**Affiliations:** Centre for Musculoskeletal Research, Menzies Health Institute Queensland, Griffith University, Gold Coast campus, QLD Australia; School of Allied Health Sciences, Griffith University, Gold Coast campus, QLD 4222 Australia; Queensland Children’s Motion Analysis Service, Children’s Health Queensland Hospital and Health Services, Brisbane, QLD Australia

**Keywords:** Gender, Lower limb, Musculoskeletal, Screening, Tolerance

## Abstract

**Background:**

The single-leg squat (SLS) test is widely used in screening for musculoskeletal injury risk. Little is known, however, of lower limb, pelvis, and trunk kinematics of SLS performance or the effect of sex and fatigue. Our aim was to determine sex differences and the influence of fatigue on SLS kinematics in healthy young adults.

**Methods:**

We recruited 60 healthy men and women between the ages of 20 and 40 years. Three-dimensional kinematic data was collected for SLSs with a ten-camera VICON motion analysis system (Oxford Metrics, UK) before and after a lower limb fatiguing exercise regime. One-way ANCOVA was used to make sex comparisons of kinematic parameters and repeated measures ANOVA was used to determine the effect of fatigue and the interaction with sex.

**Results:**

30 men (25.6 ± 4.8 years) and 30 women (25.1 ± 3.8 years) volunteered to participate. Peak pelvic rotation (3.9 ± 4.1 vs. 7.7 ± 6.2 deg, *P* = 0.03), peak hip internal rotation (−1.8 ± 5.7 vs. 3.0 ± 7.3 deg, *P* = 0.02), hip adduction range (11.7 ± 4.8 vs. 18.3 ± 6.7 deg, *P* = 0.004), and hip rotation range (10.7 ± 3.9 vs. 13.0 ± 4.2 deg, *P* = 0.04) were smaller for men than for women. Likewise, distance of mediolateral knee motion (180 ± 51 vs. 227 ± 50 mm, *P* = 0.001) was shorter for men than for women. The kinematic response to fatigue was an increase in trunk flexion, lateral flexion and rotation, an increase in pelvic tilt, obliquity and rotation, and an increase in hip flexion and adduction range (*P* ≤0.05).

**Conclusions:**

Sex differences in SLS kinematics appear to apply only at the hip, knee, and pelvis and not at the trunk. Fatiguing exercise, however, produces changes at the trunk and pelvis with little effect on the knee.

## Background

There is some evidence to suggest that certain risky lower limb movement patterns during athletic activities increase the likelihood of lower limb injuries. For example, an increase in knee valgus angle is associated with increased risk of non-contact anterior cruciate ligament (ACL) tears [1, 2]. Interestingly, the prevalence of such risky movement patterns appears to be greater amongst women than it is for men; an observation that is commensurate with ACL injury rates [3]. Laboratory-based findings show a pattern of increased peak values for knee abduction, hip adduction and internal rotation, and pelvic rotation during athletic tasks in women compared with men [4–7], suggesting that such ‘excessive’ movements may relate to the higher number of injuries sustained.

Neuromuscular fatigue, defined as an exercise-induced reduction in the maximal voluntary force produced by a muscle or group of muscles [8] is proposed to increase the risk of ACL injury via altered lower limb joint kinematics associated with poor muscle activation patterns [9]. For example, neuromuscular fatigue has been demonstrated to induce changes in knee flexion, knee abduction and hip internal rotation in female NCAA athletes performing single-leg landings [10]. Similarly undesirable kinematic patterns have been reported for young men with and without ACL reconstruction following a generalised fatigue protocol [11]. Thus, the effect of fatigue on movement patterns in common musculoskeletal screening tests is an important first step to inform injury screening and guide further injury prevention research.

Widespread clinical use of the single-leg squat test (SLS) in screening for musculoskeletal injury risk and assessment of the lower limb has generated research interest in the underlying biomechanics of the task. For instance, hip muscle weakness is associated with excessive frontal plane knee motion during the SLS [12] and kinematic differences at the hip and knee during weight-bearing activities have been reported for participants with and without patellofemoral pain syndrome [13]. Indeed, clinician ratings of SLS performance are sensitive to kinematics at the hip and knee [14] and there is high inter- and intra-rater reliability for the test [15, 16]. While kinematic examinations of the lower limb have been undertaken for a variety of tasks, few studies have considered the impact of the trunk and pelvis as a potential proximal controller [17]. Accordingly, a thorough, adequately-powered investigation of the sex differences in three-dimensional kinematics of the SLS that includes analysis of pelvic and trunk motion, is not available in the literature. This is a crucial gap in the literature as the trunk, due to its large relative mass, has the potential to substantially alter the position of the body’s centre of mass and subsequent loading of lower limb joints [18]. Furthermore, the effect of fatigue on SLS kinematics has not been reported and findings may help establish the impact of the trunk under such challenging conditions.

Our aim was to determine sex differences in three-dimensional kinematics at the trunk, pelvis, hip, and knee for the SLS task and the effect of a fatiguing exercise protocol on these parameters. We hypothesised that: 1) women would exhibit greater motion at the trunk, pelvis, hip and knee during the SLS task; 2) after fatiguing exercise, greater motion would be observed at the hip and knee; and 3) men and women would have a similar kinematic response to the fatiguing exercise protocol.

## Methods

### Study design

A controlled laboratory (pre-test post-test) study design was used to compare three-dimensional kinematic data for SLS performances between men and women and before and after a regime of fatiguing lower limb exercise.

### Participants

We recruited 60 healthy, young adults (30 men and 30 women) between the ages of 18 and 40 years (mean age 25.3 ± 4.3 years) to participate in the study. Men were 1.78 ± 0.08 m tall, weighed 77.3 ± 12.0 kg, and had a BMI of 24.3 ± 3.1 kg/m^2^. Women were 1.68 ± 0.06 m tall, weighed 59.8 ± 8.2 kg, and had a BMI of 21.2 ± 2.3 kg/m^2^. Overall, participants were considered to be moderately active with a median score of five (range, 3–9) on the Tegner Activity Level Scale [19]. Participants were included if they were generally healthy, ambulant and within the age range. Participants were excluded if they had any of the following: current or recent (past 6 months) musculoskeletal or orthopaedic injury, lower limb pain, balance or co-ordination impairment, were taking medications known to influence balance, or had a medical condition incompatible with a moderate intensity exercise bout of up to 45 min. Though we did not exclude on the basis of race, all participants were Caucasian.

Ethical approval was granted by the Griffith University Human Research Ethics Committee (Approval number: PES-27-11-HREC) and the study met the ethical standards of the journal [20]. Each volunteer gave individual written consent prior to participating and the rights of participants were protected.

### Experimental setup

A ten-camera VICON motion analysis system (MX13 Cameras, Oxford Metrics, Oxford, UK) operating at 200 Hz was used to collect three-dimensional marker trajectories attached to specific anatomical landmarks including the second metatarsal head, medial and lateral malleoli, calcaneus, medial and lateral femoral condyles, right and left anterior superior and posterior superior iliac spines, spinous process of the twelfth thoracic vertebra, spinous process of the seventh cervical vertebra, the manubrium, and the xiphoid process. Clusters of four markers were additionally attached to the shank and thigh segments. Standards indicate maximum absolute error of approximately 1 mm for reconstruction of three-dimensional marker displacements and a root-mean square error of 1.4° for three-dimensional angles. All markers were maintained throughout the exercise protocol to maintain consistency for pre and post SLS trials. To establish neutral joint positions, determine additional anatomical landmarks, and define lower limb joint coordinate systems, a series of subject calibration trials was performed. Calibration trials required subjects to stand in the anatomical position as well as perform hip and knee joint movement tasks [21, 22]. The hip joint functional movement task involved standing on one leg and performing a hip swinger trial, whereby the participant abducted and adducted their hip in varying degrees of hip flexion and extension [23]. The knee joint functional movement task involved the performance of repeated squats through the available range of knee flexion. In performing such movements, hip joint centres are identified by fitting a sphere to the motion of the thigh markers and knee joint flexion extension axes are identified by using a mean helical axis method [21, 24].

Raw three-dimensional coordinate data were filtered using a zero-lag fourth-order low-pass Butterworth filter, with a cut-off frequency of 8 Hz. Filtered marker trajectories were used to compute three-dimensional segment (trunk and pelvis) and joint (hip and knee) kinematics using BodyBuilder modelling software, version 3.6 (Vicon; Oxford Metrics). The convention used to describe joint kinematics was in accordance with the recommendations of the International Society of Biomechanics [23, 25, 26]. Segment and joint angles were calculated using the Euler angle method in a flexion/extension, abduction/adduction, and internal/external rotation sequence.

### Experimental protocol

Prior to testing, each participant completed a standard 5-minute warm-up on a cycle ergometer (Ergomedic 818E, Monark, Sweden) at 50 W (60 RPM). After the warm-up, each participant rested for approximately 5 min while camera set up was finalised and marker placement was checked. Participants were then allowed one successful SLS practice attempt prior to performing three separate individual SLSs for analysis. The leg chosen was determined by coin toss. For each SLS, participants were instructed to stand on one leg with the opposite knee flexed to approximately 90°, opposite hip in a neutral position, and hands positioned on their waist slightly above their anterior superior iliac spines. While looking straight ahead, participants were instructed to ‘squat down’ so as to achieve knee flexion of between 75 and 85° indicated by an audible tone. This range was based on the self-selected knee flexion angle observed in a previous study with similar subject demographics [15]. The knee flexion angle was streamed in live mode from the Vicon Nexus software and the audible tone was setup as a threshold level. The heel of the stance leg was allowed to rise from the floor during the SLS if necessary.

After baseline SLSs were performed and following a 5-minute rest period, each participant performed a test for maximal vertical jump height measured with a Yardstick vertical jump device (Swift Sports, Lismore, Australia). Participants began by standing on the force plate immediately adjacent to the Yardstick with their feet shoulder-width apart and looking straight ahead. The preferred arm was raised without trunk lean or scapula elevation to touch the pegs on the Yardstick, while the hand of the non-preferred arm was held by their side. The height of the reach determined the baseline height for the participant. Participants were instructed to maintain the starting position, before performing a maximal vertical jump in counter-movement fashion to strike the pegs as high as possible on the Yardstick with the hand of the preferred arm. Participants were each given five attempts and the baseline height was subtracted from the highest peg achieved to calculate maximal vertical jump height.

A general fatigue protocol was initiated, whereby each participant performed sets of lunges alternating between the test leg and the non-test leg. A maximal vertical jump was performed after each set in order to monitor any decrement in maximal jump performance. Twenty repetitions of lunges were performed for the initial three sets before incrementing by an additional 10 lunges for every set thereafter. General fatigue was defined as the culmination of both central and peripheral fatigue mechanisms inclusive of the muscular, cardiovascular and respiratory systems. Our lunging regime was designed to emulate (in a controlled manner) a pattern of lower limb exercise that might be experienced in the sporting or recreational context. As per Webster and colleagues [11], participants were declared fatigued when either their maximal jump height had diminished by 20 %, or if the participant could no longer continue to perform the lunges. Once the participant had satisfied the fatigue criteria, an additional three individual SLSs were performed in the same fashion as the first for comparison. In an effort to avoid deliberate cessation or continuation, we did not inform participants of the criteria used to determine fatigue.

### Statistics

Statistical analyses were performed using the Statistical Package for the Social Sciences (SPSS) version 21.0 for Windows (IBM, Chicago, IL, USA). Descriptive statistics, were generated for age, height, weight and BMI for the whole sample. Multivariate ANCOVA with height as a covariate was used to determine sex differences in joint kinematics in SLS performance prior to lunging exercise. Repeated measures ANOVA was employed to determine the effect of fatigue (i.e. time) on kinematic parameters and the interaction with sex. The following kinematic parameters served as dependent variables for both comparisons: trunk flexion, trunk lateral flexion, trunk rotation, pelvic tilt, pelvic obliquity, pelvic rotation, hip adduction, hip internal rotation, hip adduction range, hip rotation range, knee mediolateral distance (i.e. absolute distance travelled in the mediolateral plane irrespective of direction), and knee mediolateral displacement. Kinematic data was temporally normalised and presented as ensemble plots using pooled data from all participants. The start (0 %) and end (100 %) of the SLS was defined as a 2-degree change in knee flexion angle from the starting position.

*A priori* power analysis indicated that a sample size of 50 (25 men and 25 women) was required to achieve 80 % statistical power to detect mean between-group differences in kinematic parameters of 6.0° with a standard deviation of 8.0° and alpha level of 0.05 per previous work [15].

## Results

### Subject characteristics

There was no difference in age between men and women (25.6 ± 4.8 vs. 25.1 ± 3.8 years, *P* = 0.29); however, men were taller (1.78 ± 0.08 vs. 1.68 ± 0.06 m, *P* = 0.001), heavier (77.3 ± 12.0 vs. 59.8 ± 8.2 kg, *P* = 0.001), and had greater BMI (24.3 ± 3.1 vs. 21.2 ± 2.3 kg/m^2^, *P* = 0.001) than women.

### Fatigue protocol

Men completed 393 ± 247 lunges (range: 60 – 1,020), while women completed 274 ± 114 lunges (range: 130 – 580). Thirty-eight (63 %) subjects reached fatigue due to an inability to continue the exercise protocol. The remaining 22 (37 %) subjects experienced a 20 % reduction in maximal vertical jump height and thus, the protocol was ceased. No adverse effects were experienced by participants as a result of the exercise protocol.

### Effect of sex

**Fig. 1:**
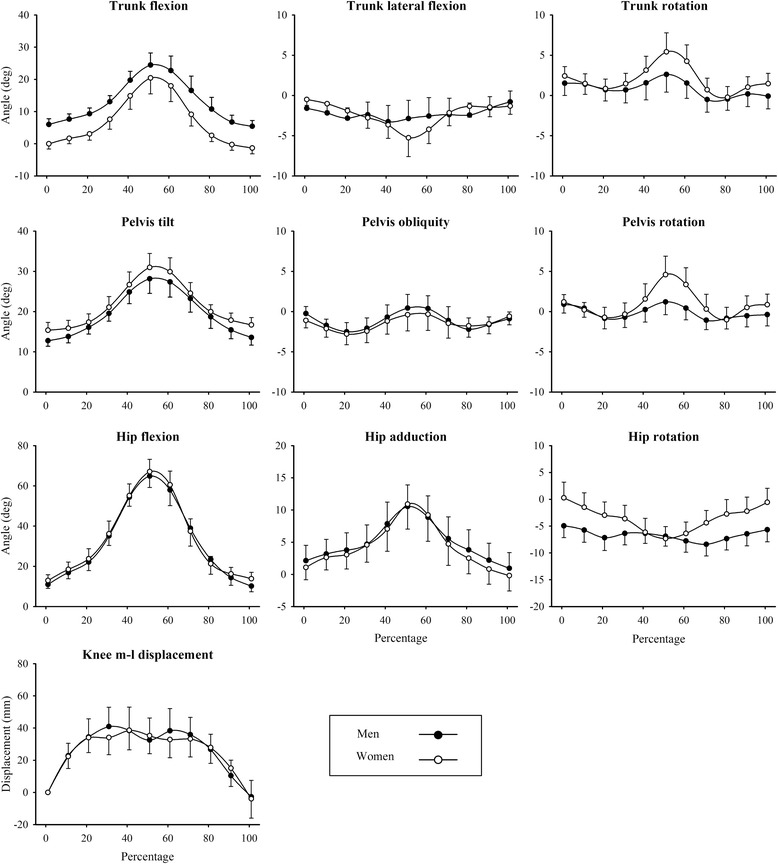
Ensemble averages for three-dimensional joint kinematics for single-leg squat performances for men and women (*n* = 60)

A main effect of sex on joint kinematics was observed for the multivariate ANCOVA conducted on pre-exercise SLSs (*F* = 2.6, *P* = 0.01). Pairwise comparisons showed no statistical differences in peak angular displacements for men and women at the trunk, however differences were apparent at the pelvis, hip and knee (*P* ≤0.05) (Table 1). At the pelvis, peak pelvic rotation (around the vertical axis) toward the stance leg (e.g. clockwise rotation for a right stance leg) was greater for women than for men (7.7 ± 6.2 vs. 3.9 ± 4.1°, *P* = 0.03). At the hip, only peak internal rotation differed between the sexes with female values greater than male values (3.0 ± 7.3 vs. -1.8 ± 5.7°, *P* = 0.02). When joint range was analysed, hip adduction range (18.3 ± 6.7 vs. 11.7 ± 4.8°, *P* = 0.004) and hip rotation range (13.0 ± 4.2 vs. 10.7 ± 3.9°, *P* = 0.04) differed between the sexes, with greater values for women on both accounts. At the knee, sex differences were observed only for medio-lateral distance, where women exhibited greater values than men (227 ± 50 vs. 180 ± 51 mm, *P* = 0.001). While participants were allowed to raise their heel from the floor during the SLS if necessary, this was not observed for any performances. Kinematic profiles for men and women are presented in Fig. 1.

**Table 1:** Three-dimensional joint kinematics (mean ± SD) for single-leg squat performances (i.e. pre-fatigue) for men and women (*n* = 60)

Kinematic parameters	Men (*n* = 30)	Women (*n* = 30)	*P* value
*Trunk*			
Peak flexion (deg)	26.6 ± 11.3	23.1 ± 15.3	0.98
Peak lateral flexion towards (deg)	0.8 ± 8.3	0.5 ± 9.9	0.48
Peak lateral flexion away (deg)	−1.6 ± 3.8	−2.2 ± 3.6	0.73
Peak rotation towards (deg)	5.6 ± 4.8	8.2 ± 6.6	0.11
Peak rotation away (deg)	−3.2 ± 3.9	−2.7 ± 2.7	0.59
*Pelvis*			
Peak tilt (deg)	28.6 ± 10.5	32.8 ± 11.6	0.23
Peak obliquity towards (deg)	−0.6 ± 6.3	0.3 ± 6.0	0.95
Peak obliquity away (deg)	−3.1 ± 4.1	−4.7 ± 4.4	0.51
Peak rotation towards (deg)	3.9 ± 4.1^*^	7.7 ± 6.2	0.03
Peak rotation away (deg)	−3.8 ± 3.0	−4.0 ± 3.4	0.64
*Hip*			
Peak adduction (deg)	12.7 ± 9.7	14.4 ± 9.8	0.39
Peak internal rotation (deg)	−1.8 ± 5.7^*^	3.0 ± 7.3	0.02
Adduction range (deg)	11.7 ± 4.8^*^	18.3 ± 6.7	0.004
Rotation range (deg)	10.7 ± 3.9^*^	13.0 ± 4.2	0.04
Knee			
Medio-lateral distance (mm)	180 ± 51^*^	227 ± 50	0.001
Medio-lateral displacement (mm)	66 ± 26^*^	89 ± 28	0.01

*significant sex difference (*P* ≤ 0.05)

### Effect of fatigue

**Fig. 2:**
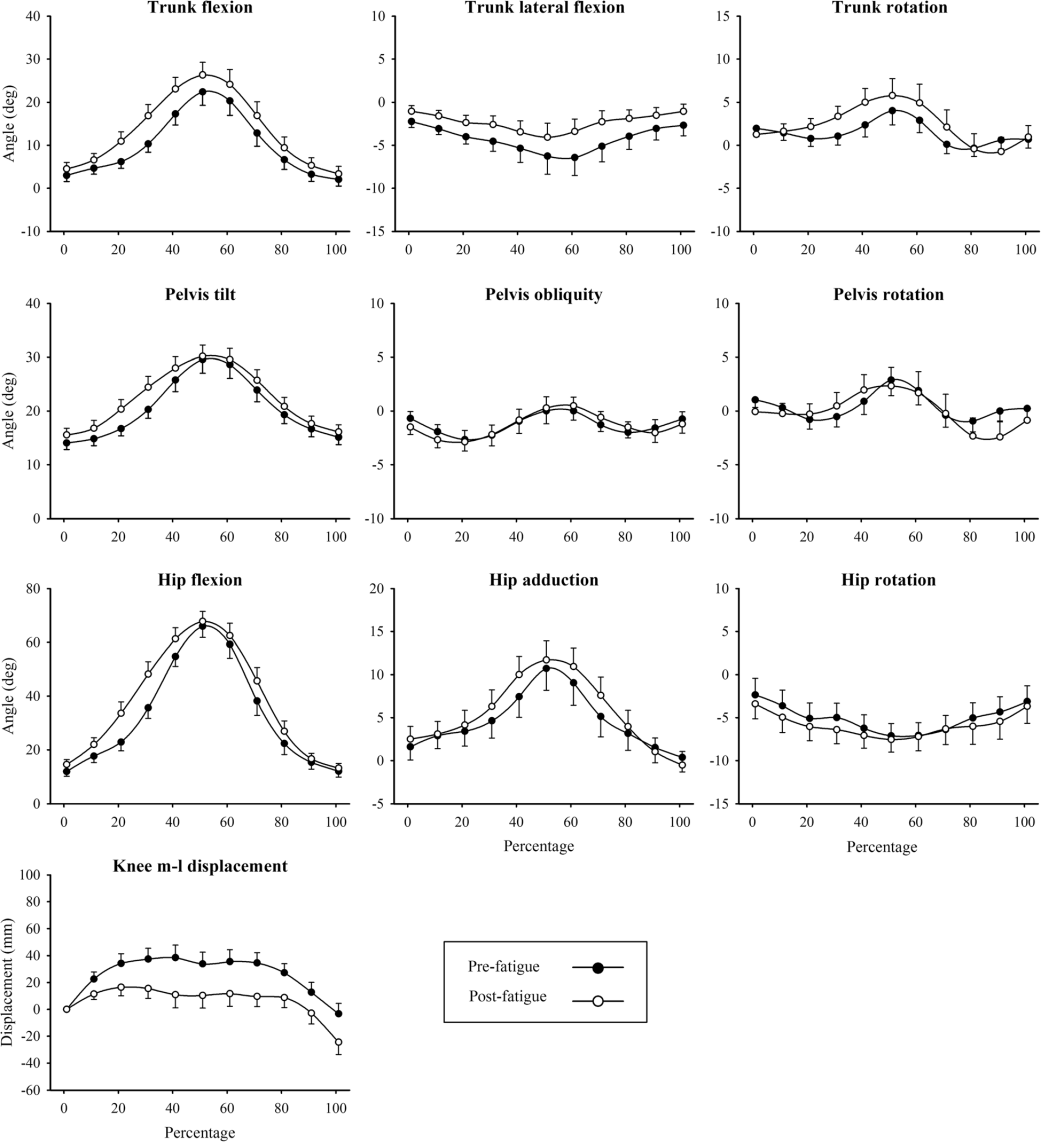
Ensemble averages for three-dimensional joint kinematics for single-leg squat performances before and after a generalised lower limb fatigue protocol (*n* = 60)

Repeated measures ANOVA conducted on pre- and post-exercise SLS performances showed a main effect for fatigue (*F* = 4.31, *P* = 0.001), but not for sex (*F* = 1.34, *P* = 0.24). Several within-group differences in three-dimensional joint kinematics for the SLS were observed following the fatiguing exercise protocol (Table 2). At the trunk, increases in peak flexion (24.5 ± 13.7 vs. 29.8 ± 11.8°), and both lateral flexion away (−7.0 ± 3.9 vs. -3.3 ± 13.0°) and rotation towards the stance leg (6.8 ± 5.7 vs. 10.1 ± 7.9°) were found in the fatigued state (*P* ≤0.05). At the pelvis, an increase in peak rotation away from the stance leg (−4.0 ± 3.0 vs. -5.4 ± 5.0°) and peak obliquity towards the stance leg (−5.2 ± 3.3 vs. -1.9 ± 6.9°) was exhibited (*P* ≤0.05). At the hip, an increase in adduction range was observed (15.2 ± 6.6 vs. 16.9 ± 6.4°, *P* = 0.04); however, there were no changes in peak adduction, peak rotation or rotation range (*P* >0.05). No significant changes were observed for knee joint kinematic parameters. Kinematic profiles for pre- and post-fatigue SLSs are presented in Fig. 2.

**Table 2:** Three-dimensional joint kinematics (mean ± SD) for single-leg squat performances before and after a lower limb fatigue protocol (*n* = 60)

Kinematic parameter	Pre (*n* = 60)	Post (*n* = 60)	Direction of change	*P* value
*Trunk*				
Peak flexion (deg)	24.5 ± 13.7	29.8 ± 11.8*	↑	0.001
Peak lateral flexion towards (deg)	2.3 ± 4.6	1.7 ± 4.5	–	0.42
Peak lateral flexion away (deg)	−7.0 ± 3.9	−3.3 ± 13.0*	↓	0.03
Peak rotation towards (deg)	6.8 ± 5.7	10.1 ± 7.9*	↑	0.001
Peak rotation away (deg)	−3.2 ± 3.9	−3.5 ± 4.6	–	0.67
*Pelvis*				
Peak tilt (deg)	30.4 ± 10.8	31.8 ± 8.7*	↑	0.05
Peak obliquity towards (deg)	3.7 ± 4.3	3.6 ± 4.0	–	0.86
Peak obliquity away (deg)	−5.2 ± 3.3	−1.9 ± 6.9*	↓	0.001
Peak rotation towards (deg)	5.7 ± 5.3	6.9 ± 5.4	–	0.10
Peak rotation away (deg)	−4.0 ± 3.0	−5.4 ± 5.0^*^	↑	0.04
*Hip*				
Peak adduction (deg)	13.5 ± 9.5	14.8 ± 8.6	–	0.11
Peak internal rotation (deg)	0.9 ± 6.9	1.0 ± 6.3	–	0.90
Adduction range (deg)	15.2 ± 6.6	16.9 ± 6.4*	↑	0.04
Rotation range (deg)	11.8 ± 4.1	12.4 ± 3.9	–	0.27
Knee				
Medio-lateral distance (mm)	207 ± 55	214 ± 83	–	0.52
Medio-lateral displacement (mm)	79 ± 30	75 ± 34	–	0.33

*significantly different from pre (*P* ≤0.05)

### Effect of sex and fatigue

There was no combined effect of sex and fatigue on SLS kinematics as evidenced by a lack of interaction on repeated measures ANOVA (*F* = 1.10, *P* = 0.41).

## Discussion

In addressing sex differences in SLS kinematics, we found that peak pelvic rotation toward the stance leg, peak hip internal rotation, hip adduction and rotation range, and knee mediolateral distance were greater for women than for men. Following fatiguing exercise, we observed increases in peak trunk flexion, lateral flexion, and rotation, peak pelvic tilt, obliquity, and rotation, and peak hip flexion and adduction range; however, the response was not sex-specific. Such findings reinforce the contribution of neuromuscular fatigue to undesirable lower limb movement patterns and the influence of the trunk and pelvis in the control of lower limb movement tasks [17, 27].

### Sex

The sex differences in SLS kinematics that were observed in the current study are in line with findings of other investigations and could be inferred to support proposed associations between kinematic sex differences during athletic tasks and lower limb injury risk [2, 28], however such associations were not assessed in the current study. It appears that in performing a SLS test, women exhibit a characteristic lower limb kinematic profile of greater hip rotation [7, 29], hip adduction [4, 7], and knee valgus [7, 29] compared to men. In broad terms, women tend to experience a greater ‘valgus collapse’, whereby the hip (through adduction and internal rotation) and the knee (through medial translation) deviate more so than men. In the clinical context, these larger deviations are interpreted by therapists as ‘poor’ or ‘bad’ [14]. In the current study, we additionally observed greater pelvic rotation in women than in men. Given this, long term prospective studies are needed to confirm if these SLS kinematic sex differences translate into injuries in order to confirm if neuromuscular training is indicated on this basis.

The magnitude of the observed sex differences in SLS kinematics is worthy of discussion. For example, discrepancies of 4.8° in peak hip internal rotation, 6.6° in hip adduction range, and 47 mm mediolateral knee distance between the sexes are likely to be clinically important as previous work demonstrates that changes of similar magnitudes in these parameters predict therapist ratings of SLS performance [15]. This is particularly important if the SLS test is to be incorporated in musculoskeletal screening or provide a basis for rehabilitation. For instance, Willy and Davis [30] observed a significant 5.4° change in hip internal rotation for the single-leg squat following a successful hip strengthening regime in healthy women. The clinical significance of the observed 3.8° difference in peak pelvic rotation, 2.3° difference in hip rotation range, and 23 mm difference in mediolateral knee displacement, however, is less clear and would warrant further investigation with respect to clinical populations, therapist assessment and injury incidence.

Only two other studies, including one very recent report, have investigated sex differences in trunk kinematics during the SLS. Both studies, however, found somewhat different results to those of the current study, albeit in much smaller samples [4, 7]. Graci and colleagues [4] for example, reported that women had less trunk flexion and greater trunk and pelvic rotation than men. Similarly, Zeller and colleagues [7] showed that women exhibited less trunk flexion than men during a SLS. Despite a larger sample size compared with previous reports, we did not find any sex differences at the trunk. In all, it seems important that the trunk be included in any future biomechanical investigation of athletic activities of men and women. Certainly, sex differences in kinematics are reported for other activities, such as cutting [31] and landings [32].

### Fatigue

Our study represents the first investigation of SLS performance before and after fatiguing exercise. We found that fatigue induced an increase in trunk flexion, lateral flexion, and rotation, pelvic tilt, obliquity, and rotation, and hip flexion. We did not observe any changes at the knee. Thus, fatigue resulted in larger movements of the trunk and pelvis, while pre-fatigue movements of the hip and knee were relatively well preserved. Interestingly, this kinematic pattern does not reflect the characteristic ‘poor’ pattern (or valgus collapse). Despite a lack of research on the effect of fatigue on the SLS, a number of groups have investigated the effect on other activities. Kinematic changes at the knee and hip, for example, have been observed post-fatigue for men and women performing single-leg stop jumps [33], landings [34–36], maximal repeated jumps [37], and sidestep cutting manoeuvres [38]. Measures of trunk kinematics were unfortunately not reported. Unlike those reports, the kinematic changes we observed post-fatigue were less striking at the knee and hip, but more apparent at the trunk and pelvis, suggesting that proximal movements at the trunk and pelvis were preferentially made in order to preserve upright stability, presumably to reduce the moment demand at the knee and thus the force contribution from the fatigued quadriceps muscles. Indeed, others have observed a connection between the trunk and the knee under other conditions, such as cutting and landing tasks [17, 39]. Future studies assessing force contributions of muscles pre and post fatigue are needed to confirm the mechanism of the kinematic changes observed in the current study.

While several post-fatigue kinematic measures were significantly different to pre-fatigue measures, the absolute change in some angles was relatively small, particularly for the pelvis (e.g. less than 2° for peak pelvic tilt and peak pelvic rotation). Such changes in individual kinematic parameters are unlikely to be clinically meaningful on their own. Nonetheless, when all significant parameters are considered together (i.e. 6 in total at the trunk and pelvis) they may represent an important deviation from the original performance, particularly when no changes were observed in peak hip or knee angles post-fatigue. The significant fatigue changes in peak trunk angles (i.e. 3.3–5.3°), however, are similar in magnitude to those observed for jump landings for people with patellofemoral pain syndrome (i.e. 2.3° lateral trunk flexion) [40].

The lack of interaction effect of sex and fatigue suggests that the effect of fatiguing exercise on SLS kinematics is not sex-specific, but rather a more pervasive, general response to the challenge. Furthermore, the post-fatigue kinematic pattern does not represent amplification of those kinematics considered to be ‘poor’ (e.g. increased hip adduction and internal rotation, and medio-lateral knee motion), but rather altered movement of other segments, such as the trunk and pelvis. Similarly null sex differences in kinematics post-fatigue have been presented for men and women performing bilateral landings from a jump [41], single-leg stop-jumps [33], single-leg drop landings [34], and repeated maximal jumps [37]. The lack of sex difference may be related to our choice of exercise for the fatigue protocol, and thus, it may be that different exercises may elicit divergent results for the sexes.

Several limitations warrant acknowledgement. Firstly, while our fatigue protocol was designed to reflect repeated bouts of physical activity that might be experienced in the sporting context, our tightly controlled lunging regime could be considered somewhat artificial and may not be directly transferrable. Our regime, however, represents an intermediary step to understanding the effects of fatigue on lower limb kinematics. Secondly, our cohort was healthy and free from musculoskeletal injury and thus, implications for injury or injury risk could only be inferred rather than quantified. Participants with injury (or with recent injury) may exhibit a different response to fatiguing exercise and this should be addressed in future studies. Thirdly, we present kinematic data to support our findings, whereas inclusion of kinetic data may be more informative with respect to the relative contribution of muscle forces to the pre and post kinematic pattern. Finally, most of our participants (i.e. 63 %) were deemed to be fatigued based on an inability to continue rather than the more objective vertical jump criterion. We based our criteria on those of a previous study [11], however a revised cut-off for the reduction in vertical jump height may be beneficial for future work.

## Conclusions

In conclusion, the current study represents a robust account of the three-dimensional kinematics of the SLS for men and women, including the effect of fatigue on such performance. Our findings strengthen what is known of the sex differences in lower limb kinematics and are significant given the known discrepancies in injury rates between the sexes. Furthermore, our results highlight the involvement of the trunk in carrying out lower limb movement tasks under challenging conditions, such as fatigue.
